# Supplementary material for: Genome-resolved metatranscriptomics reveals conserved root colonization determinants in a synthetic microbiota
Source: Nat Commun. 2023 Dec 13;14:8274. doi: 10.1038/s41467-023-43688-z (PMC10719396; doi:10.1038/s41467-023-43688-z)
Supplement: Supplementary file 1 — Supplementary Information [file 41467_2023_43688_MOESM1_ESM.pdf]

Supplementary information for

Genome-resolved metatranscriptomics reveals conserved root colonization determinants in a synthetic microbiota

Nathan Vannier[1,2], Fantin Mesny[1,3,*], Felix Getzke[1,*], Guillaume Chesneau[1], Laura Dethier[1], Jana Ordon[1], Thorsten Thiergart[1], Stéphane Hacquard[1,4,#]

[1]Department of Plant Microbe Interactions, Max Planck Institute for Plant Breeding Research 50829 Cologne, Germany
[2]Present address: IGEPP, INRAE, Institut Agro, Univ Rennes, 35653, Le Rheu, France
[3]Present address: Institute for Plant Sciences, University of Cologne, 50923 Cologne, Germany
[4]Cluster of Excellence on Plant Sciences, Max Planck Institute for Plant Breeding Research 50829 Cologne, Germany
*These authors contributed equally
[#]Corresponding author: S.H.: hacquard@mpipz.mpg.de

Supplementary Figures

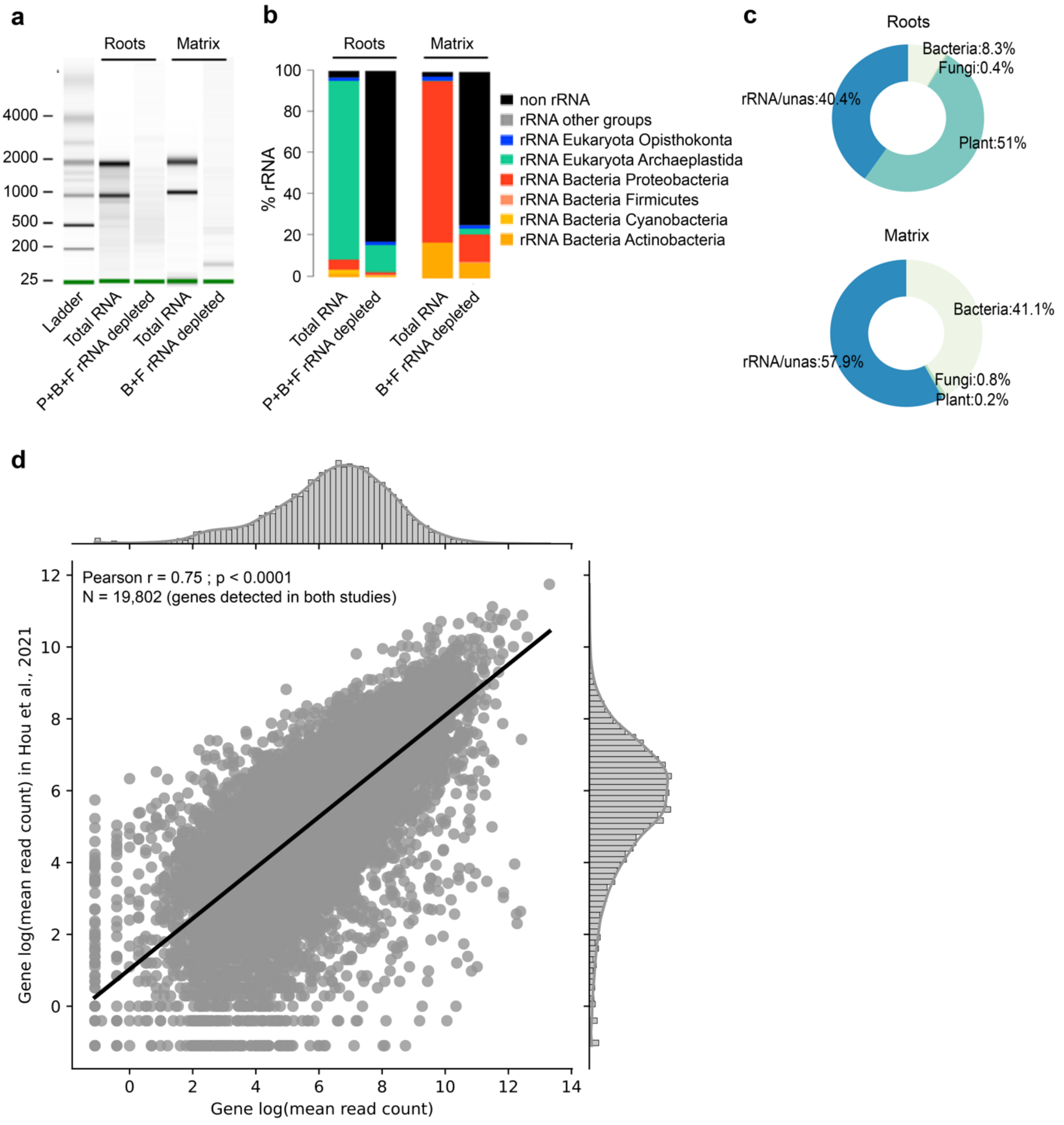

Supplementary Fig. 1: Multi-kingdom ribosomal RNA depletion and read mapping. (**a**) Validation of multi-kingdom rRNA depletion for roots and soil matrix samples with a bio-analyzer. (**b**) Validation of multi-kingdom RNA depletion for roots and soil matrix samples based on RNA-Seq read mapping. (**a**,**b**) One sample per condition is shown. P: plant, F: fungi, B: bacteria. (**c**) Proportion (%) of reads mapped to the reference genomes of plant, bacteria and fungi as well as unassigned reads in the roots (top) and peat matrix (bottom) samples, three samples per condition. (**d**) Correlation of *A. thaliana* log-transformed mean read counts in root samples between this study and a previous study (Hou et al. 2021[1], condition NC+BFO) in which plants were grown in the same gnotobiotic plant system under the same environmental conditions. Note that the SynCom used by Hou et al. 2021 was bigger (183 bacteria, 24 fungi, 7 oomycetes) but includes > 90% of the strains used in this study.

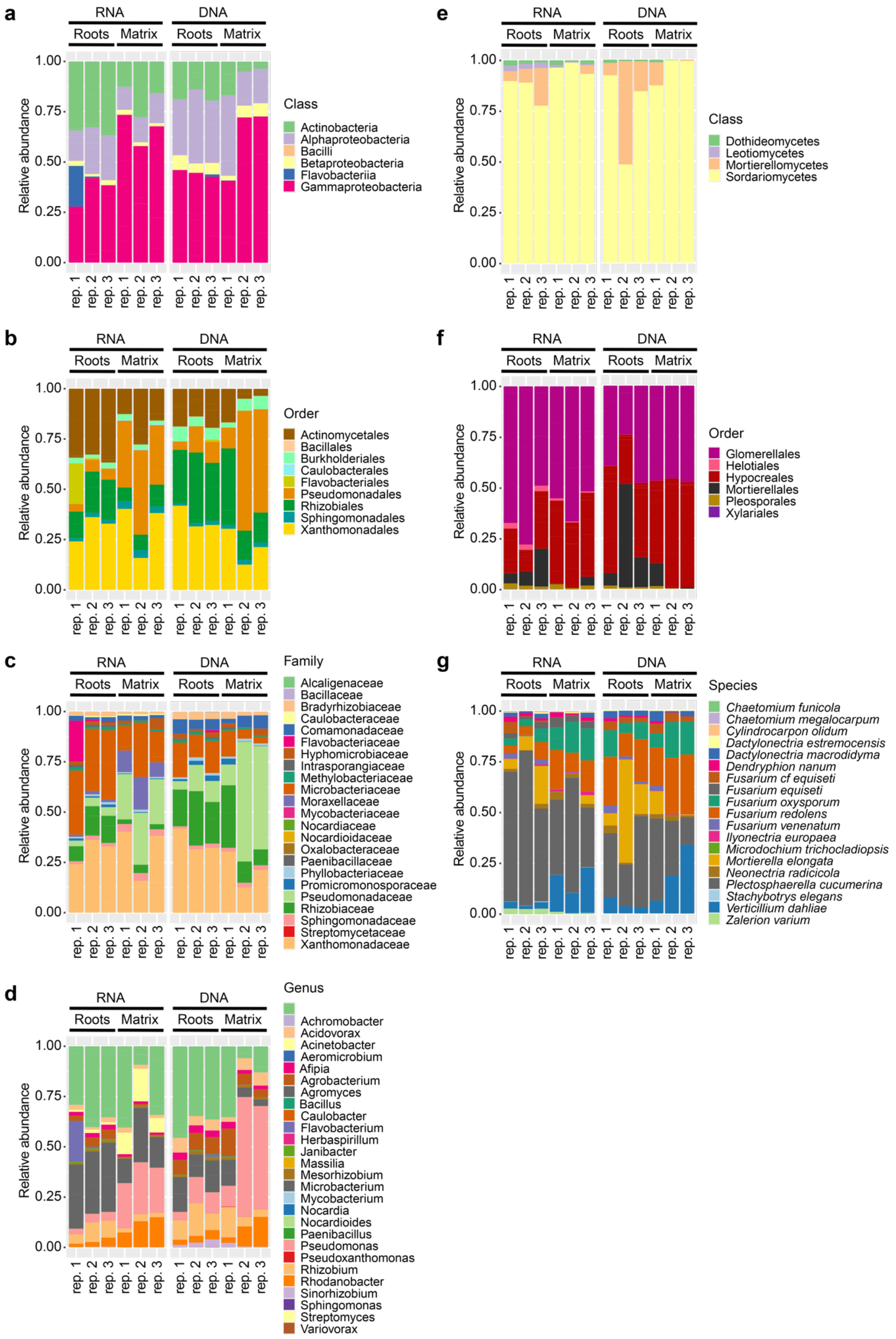
a
RNA
DNA
Roots
Matrix
Relative abundance
1.00
0.75
0.50
0.25
0.00
rep. 1
rep. 2
rep. 3
Class
Actinobacteria
Alphaproteobacteria
Bacilli
Betaproteobacteria
Flavobacteriia
Gammaproteobacteria
b
Order
Actinomycetales
Bacillales
Burkholderiales
Caulobacterales
Flavobacteriales
Pseudomonadales
Rhizobiales
Sphingomonadales
Xanthomonadales
c
Family
Alcaligenaceae
Bacillaceae
Bradyrhizobiaceae
Caulobacteraceae
Comamonadaceae
Flavobacteriaceae
Hyphomicrobiaceae
Intrasporangiaceae
Methylobacteriaceae
Microbacteriaceae
Moraxellaceae
Mycobacteriaceae
Nocardiaceae
Nocardioidaceae
Oxalobacteraceae
Paenibacillaceae
Phyllobacteriaceae
Promicromonosporaceae
Pseudomonadaceae
Rhizobiaceae
Sphingomonadaceae
Streptomycetaceae
Xanthomonadaceae
d
Genus
Achromobacter
Acidovorax
Acinetobacter
Aeromicrobium
Afipia
Agrobacterium
Agromyces
Bacillus
Caulobacter
Flavobacterium
Herbaspirillum
Janibacter
Massilia
Mesorhizobium
Microbacterium
Mycobacterium
Nocardia
Nocardioides
Paenibacillus
Pseudomonas
Pseudoxanthomonas
Rhizobium
Rhodanobacter
Sinorhizobium
Sphingomonas
Streptomyces
Variovorax
e
Class
Dothideomycetes
Leotiomycetes
Mortierellomycetes
Sordariomycetes
f
Order
Glomerellales
Helotiales
Hypocreales
Mortierellales
Pleosporales
Xylariales
g
Species
Chaetomium funicola
Chaetomium megalocarpum
Cylindrocarpon olidum
Dactylonectria estremocensis
Dactylonectria macrodidyma
Dendryphion nanum
Fusarium cf equiseti
Fusarium equiseti
Fusarium oxysporum
Fusarium redolens
Fusarium venenatum
Ilyonectria europaea
Microdochium trichocladiopsis
Mortierella elongata
Neonectria radicicola
Plectosphaerella cucumerina
Stachybotrys elegans
Verticillium dahliae
Zalerion varium

Supplementary Fig. 2: Comparison between RNA-based and DNA-based SynCom profiling. (**a**-**g**) Relative abundance profiles of bacterial (**a-d**) and fungal (**e-g**) strains (y-axis) in roots and matrix samples using RNA-based (RNA, left) and DNA-based (DNA, right) profiling. Strains' RAs were aggregated at the class (**a**), order (**b**) family (**c**) and genus (**d**) levels for bacteria and at the class (**e**), order (**f**) and species (**g**) levels for fungi (n = 3 replicates for each condition). Source data are provided as a Source Data file.

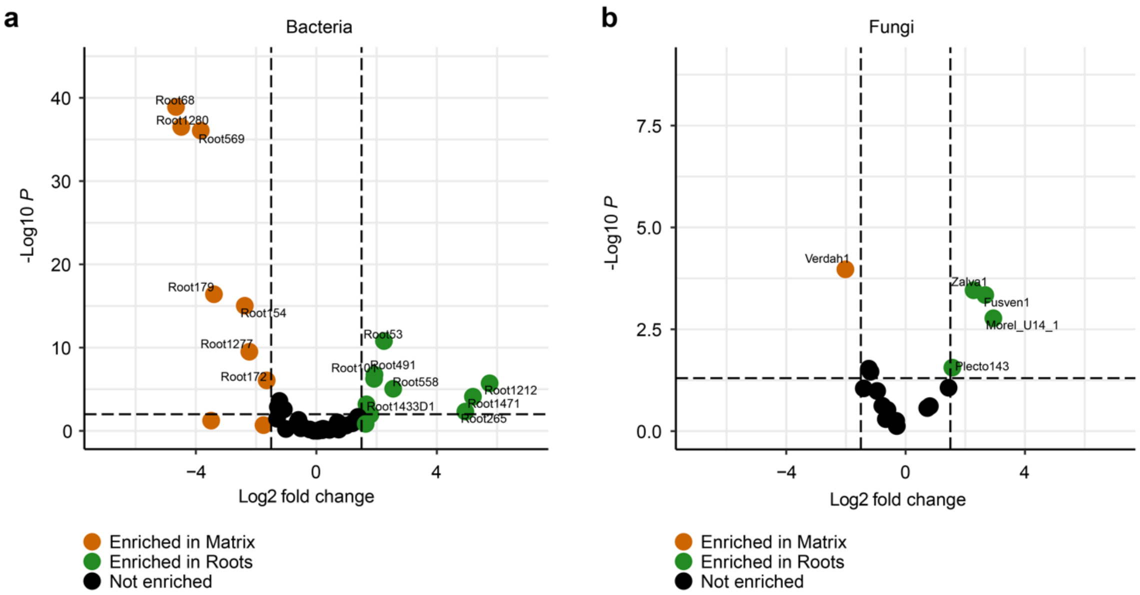

Supplementary Fig. 3: Bacterial and fungal strains enrichment between roots and matrix samples. Volcano plot showing the log2FC and *p*-value of bacterial (**a**) and fungal (**b**) abundance in roots compared to soil matrix. Enrichment tests were done using DESeq2[2] with RNA reads counts per strain. Source data are provided as a Source Data file.

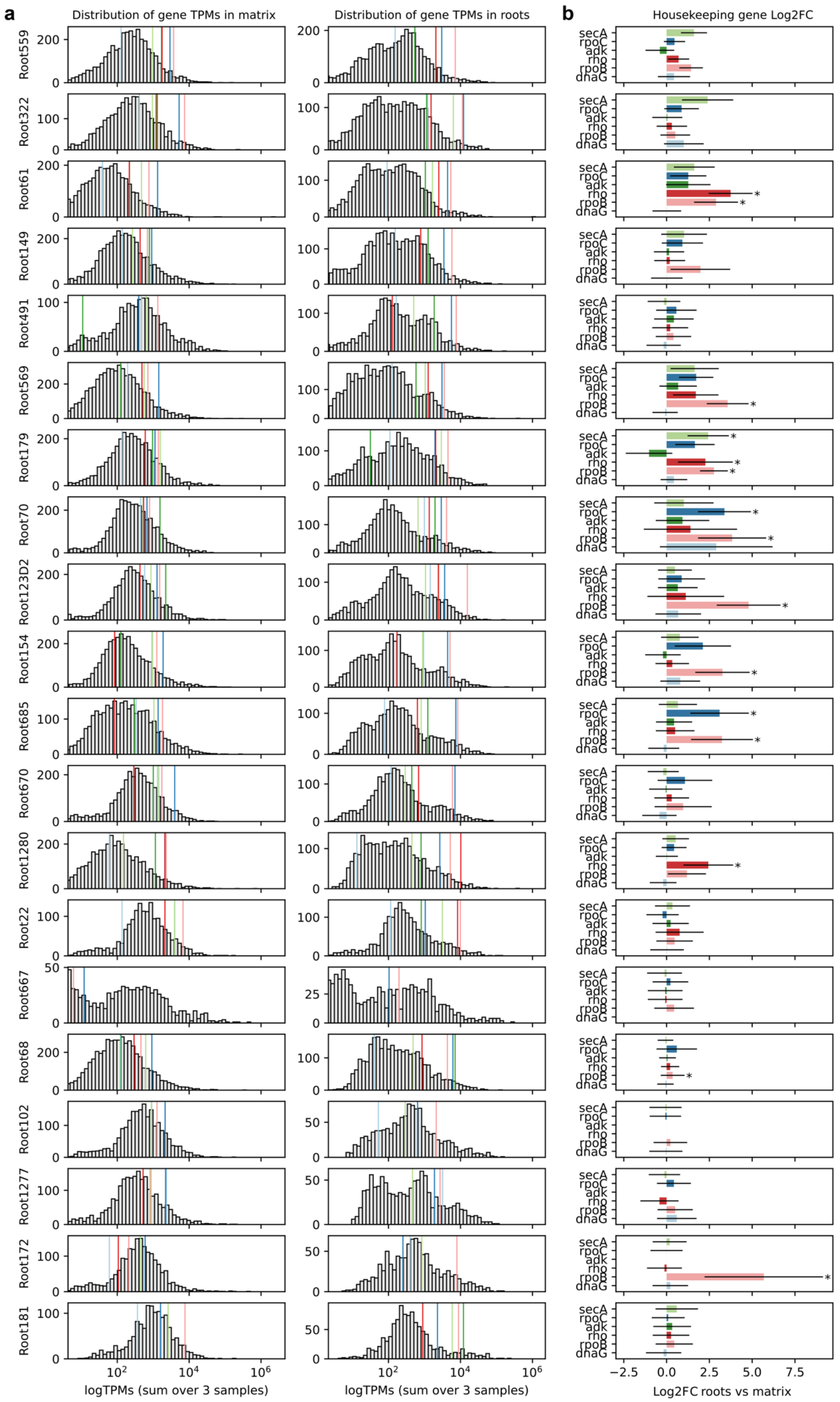
a
Distribution of gene TPMs in matrix
Distribution of gene TPMs in roots
b
Housekeeping gene Log2FC
Root559
Root322
Root61
Root149
Root491
Root569
Root179
Root70
Root123D2
Root154
Root685
Root670
Root1280
Root22
Root667
Root68
Root102
Root1277
Root172
Root181
secA
rpoC
adk
rho
rpoB
dnaG
logTPMs (sum over 3 samples)
logTPMs (sum over 3 samples)
Log2FC roots vs matrix

Supplementary Fig. 4: Quality assessment of the 20 bacterial transcriptomes selected for further analysis. (**a**) Distribution of the per-strain log-transformed Transcripts Per Kilobase Million (TPM) values of bacterial detected genes, in matrix (left) and roots (right) independently. Nearly all transcriptomes show normal or quasi-normal distribution, revealing that our pseudo-mapping results are suited to differential expression analyses. (**b**) Log2FC values of six reference bacterial housekeeping genes calculated by DESeq2[2] differential expression analysis. These genes were selected as (1) they were annotated by emapper[3] in our bacterial genomes; (2) they are present in a single copy in our reference genomes (no paralog); (3) their expression has been characterized to be invariable in more than 80% of the studies according to considered in the meta-analysis performed by Rocha et al. (2015)[4]. Genes identified as significantly differentially expressed (adjusted *p*-value < 0.05) are marked with an asterisk. The TPM value of these genes is marked on the histograms of panel a by vertical bars using the same color code. Source data are provided as a Source Data file.

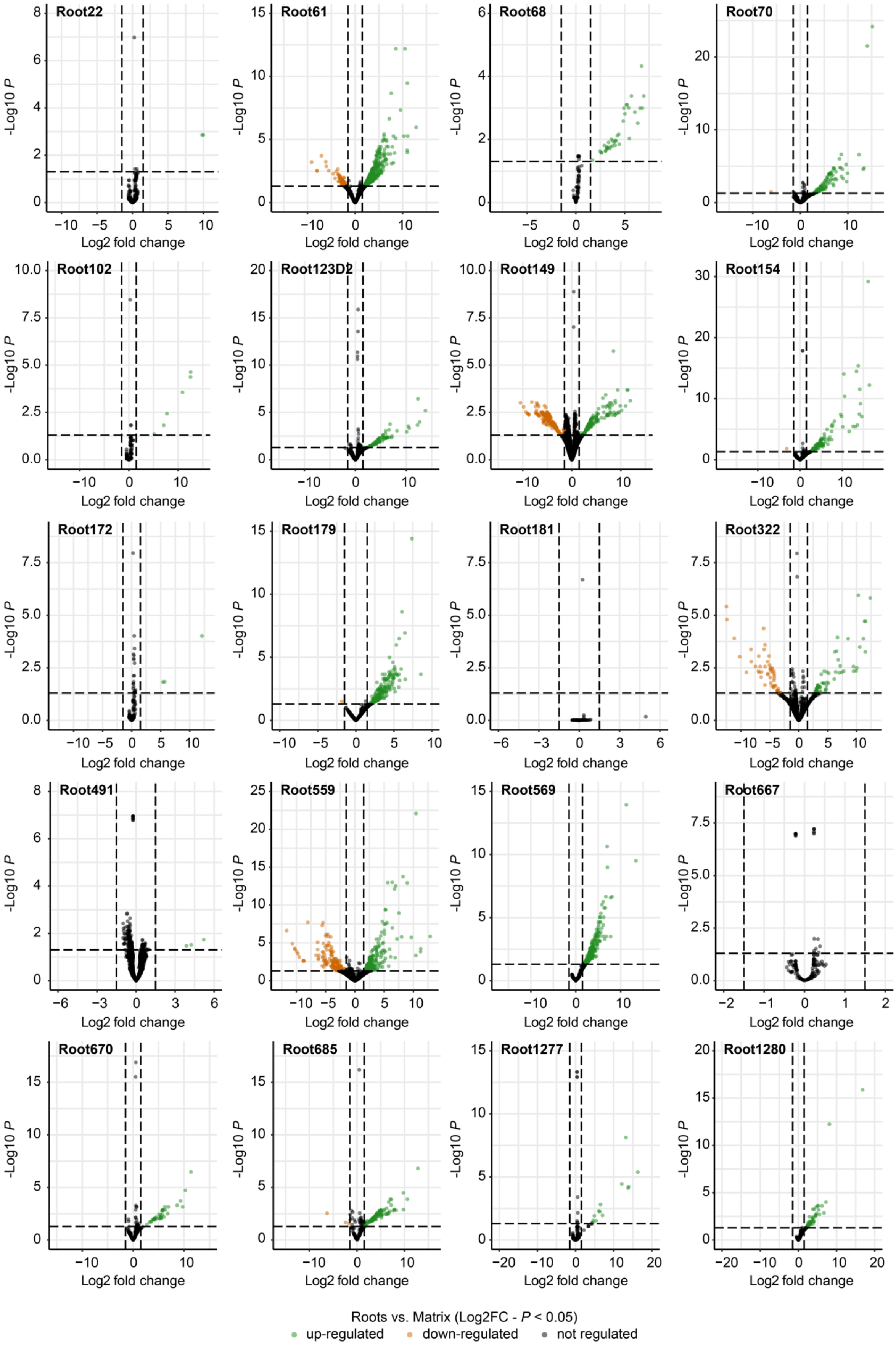
Root22
Root61
Root68
Root70
Root102
Root123D2
Root149
Root154
Root172
Root179
Root181
Root322
Root491
Root559
Root569
Root667
Root670
Root685
Root1277
Root1280
-Log10 P
Log2 fold change
Roots vs. Matrix (Log2FC - P < 0.05)
up-regulated
down-regulated
not regulated

Supplementary Fig. 5: Top 20 bacterial strains differential gene expression in roots. Volcano plots showing DESeq2[2] log2FC and *p*-value for gene´s transcripts enrichment in roots compared to soil matrix. Transcripts significantly enriched (adjusted $P < 0.05$) are highlighted in color with transcripts significantly enriched in roots in green (log2FC > 1.5) and transcripts significantly enriched in soil matrix in orange (log2FC < -1.5). Source data are provided as a Source Data file.

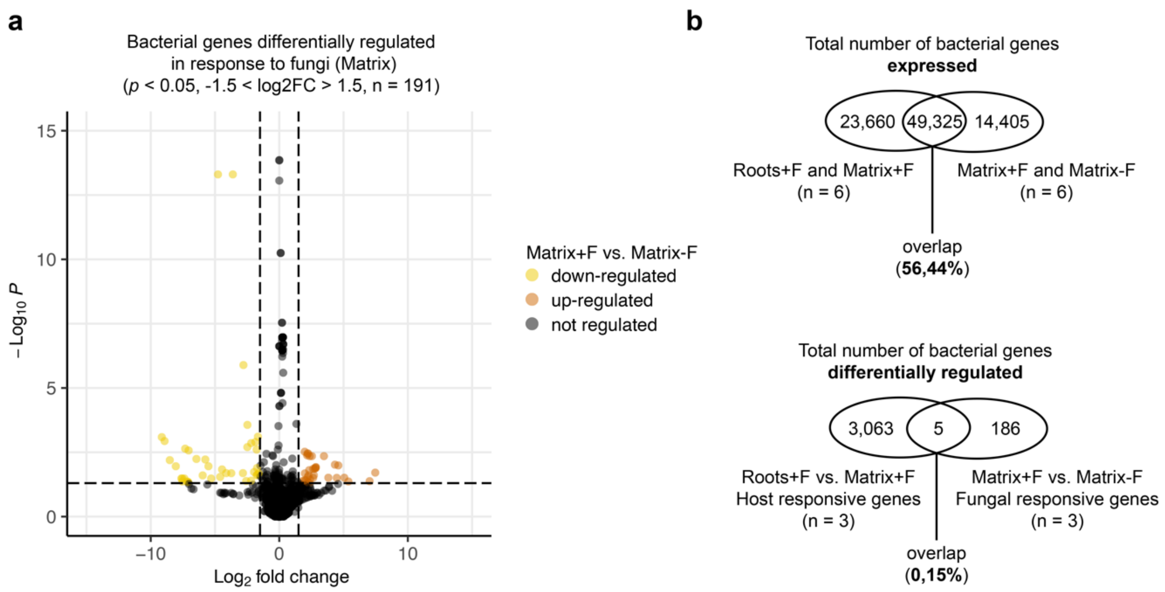

Supplementary Fig. 6: **Comparison of bacterial gene sets regulated in response to host and fungal presence.** (**a**) Volcano plot depicting bacterial genes differentially expressed (DEGs) in presence vs. absence of the 22-member fungal SynCom (FlowPot system, 4 weeks post inoculation) in a second experiment performed in matrix samples with the 84-member bacterial SynCom (Matrix+F vs. Matrix-F, n = 3 replicates each, DESeq2, adjusted $P < 0.05$). The 191 DEGs highlighted in color are significantly down- (yellow, n = 40) or up- (orange, n = 32) regulated in presence vs. absence of fungi (-1.5 < log2FC > 1.5). (**b**) Venn diagrams comparing the total number of expressed genes (upper panel, at least one transcript in one sample) or DEGs (lower panel, DESeq2[2], adjusted $P < 0.05$, -1.5 < log2FC > 1.5) between two independent bacterial transcriptome datasets. The first dataset corresponds to the main bacterial transcriptome data involving roots and matrix samples in the presence of fungi (Roots+F, Matrix+F, bacterial response to the host in the presence of fungi) (see also **Fig. 2a**, and **Supplementary Table 2**), whereas the second dataset corresponds to the second above-mentioned bacterial transcriptome data from matrix samples in presence vs. absence of the fungal SynCom (Matrix+F, Matrix-F, bacterial response to fungi in the matrix, see panel **a**).

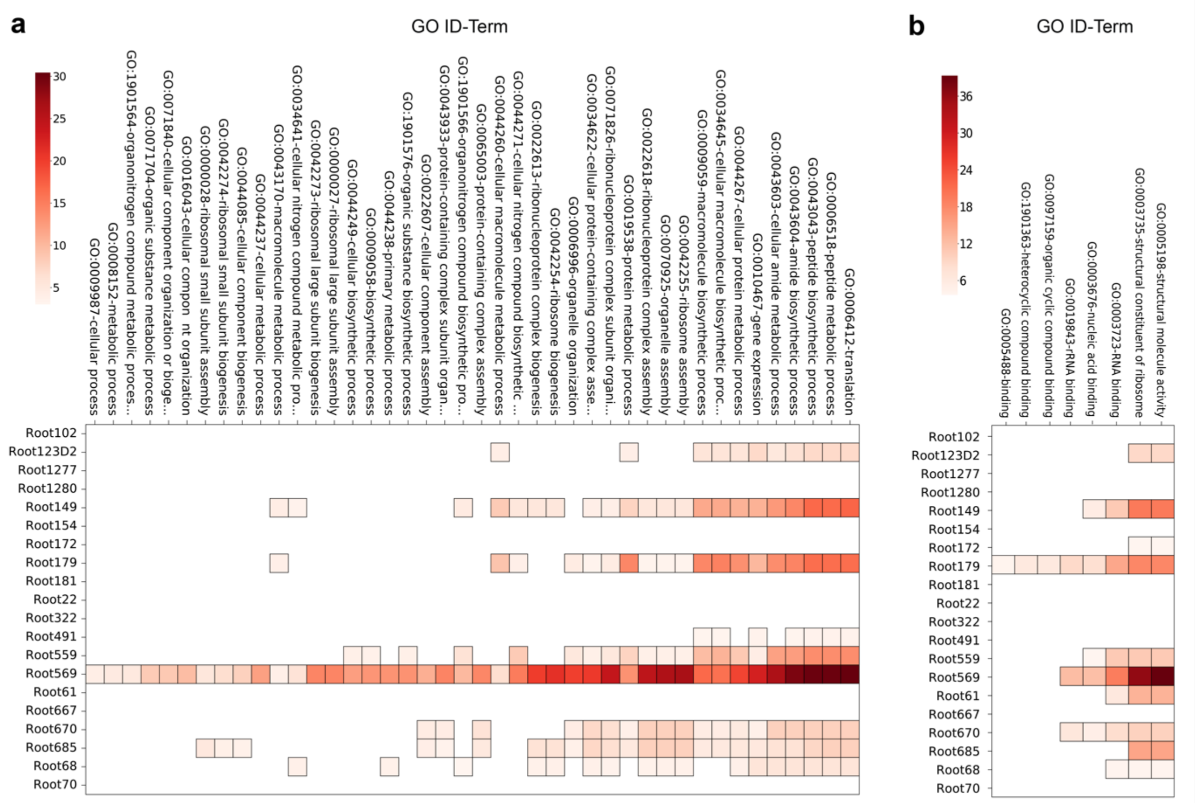

Supplementary Fig. 7: Individual GO terms enrichment analyses of the top 20 bacterial strains. GO terms enrichment testing for biological process (**a**) and molecular function (**b**) calculated from individual strains transcriptomes. Colors indicate the adjusted *p*-value for the enrichment testing which indicates the probability of finding the observed number of up-regulated genes with a given function (compared to random sampling of genes from the strain's genome) according to Fisher's exact test (as implemented in topGO[5]). Source data are provided as a Source Data file.

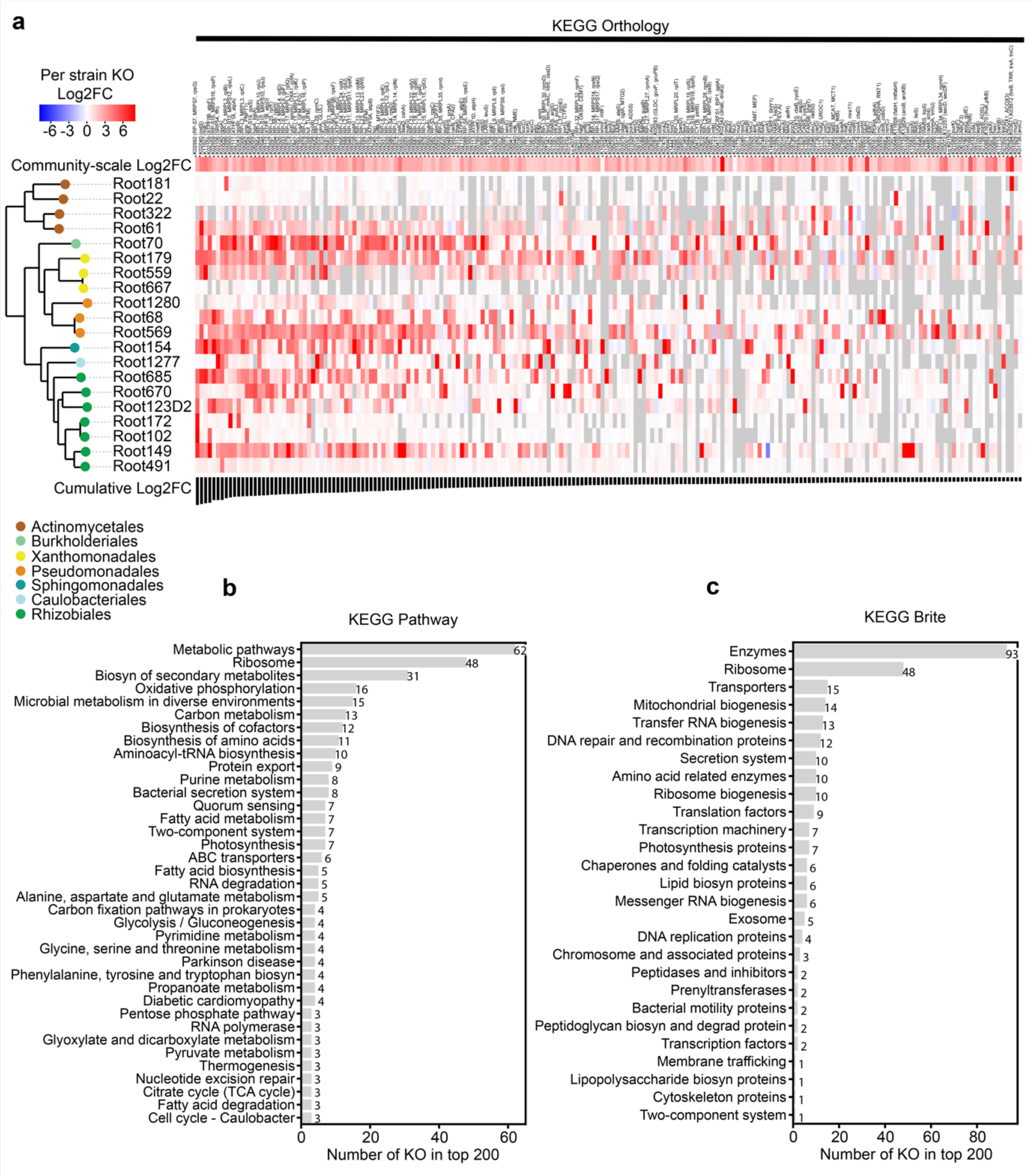

Supplementary Fig. 8: Regulation of functions in bacteria in response to roots. (**a**) Heatmap showing 200 KEGG[6] terms differential expression (i.e., pooled transcripts count per KEGG orthology term) in roots compared to soil matrix across the 20 most covered strains. KEGG terms presented are the top 200 with the highest cumulative log2FC across the 20 bacterial strains and are ordered based on this cumulative log2FC. The names and annotation (when annotated) of KEGG terms are indicated (top). The log2FC at the community scale (calculated by pooling transcripts counts from identical KEGG terms between strains) are indicated (upper row) as well as a phylogenomic tree (method STAG from OrthoFinder[3]) for the 20 bacterial strains (left) and RNA-based relative abundances of strains in the roots samples (right). (**b**) Number of KEGG terms of KEGG pathways found in the top 200 KEGG terms. (**c**) Number of KEGG terms of KEGG Brite found in the top 200 KEGG terms. Source data are provided as a Source Data file.

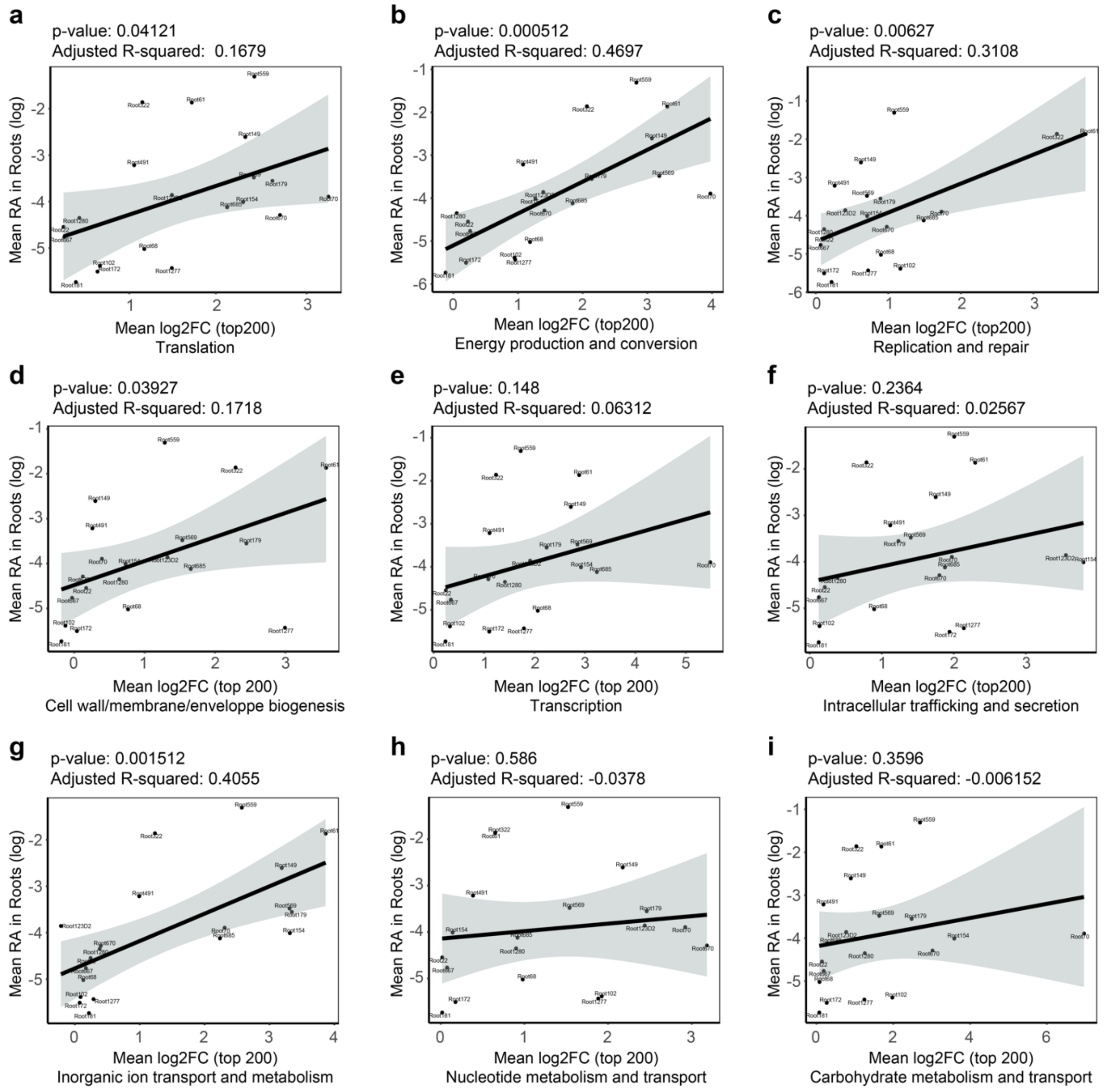

Supplementary Fig. 9: Correlation between function regulation and strains relative abundance in roots. (**a-i**) Linear regressions between average function regulation in roots and strains relative abundance for different functions. Function regulation is calculated as the average log2FC of all orthogroups (OGs) annotated as a given function within the top 200 OGs. Strains' abundance is calculated as the RNA-based relative abundance. Indicated *p*-values and R^2 are obtained from a linear regression. Source data are provided as a Source Data file.

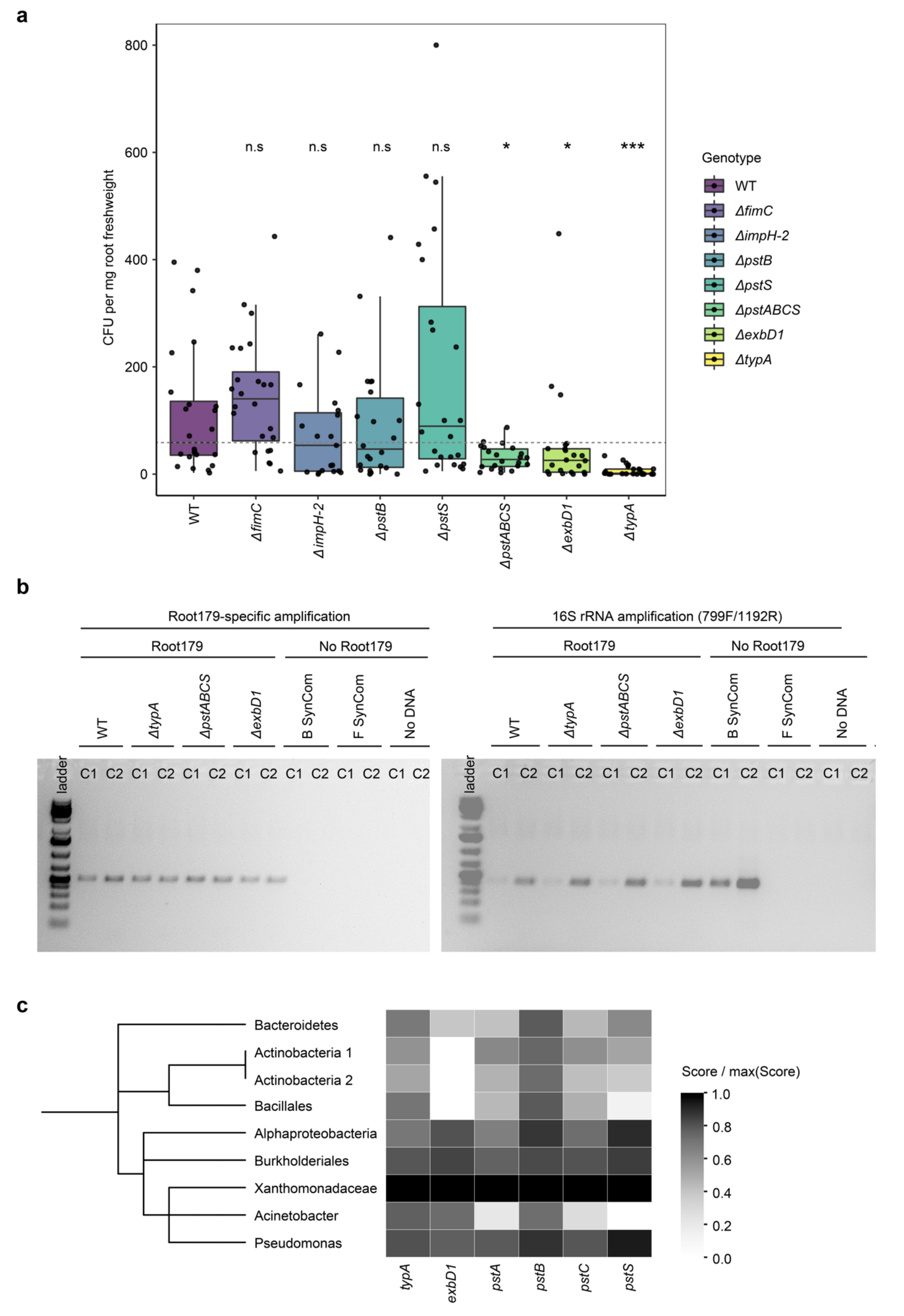
a
CFU per mg root freshweight
800
600
400
200
0
n.s
n.s
n.s
n.s
*
*
***
WT
ΔfimC
ΔimpH-2
ΔpstB
ΔpstS
ΔpstABCS
ΔexbD1
ΔtypA
Genotype
WT
ΔfimC
ΔimpH-2
ΔpstB
ΔpstS
ΔpstABCS
ΔexbD1
ΔtypA
b
Root179-specific amplification
Root179
No Root179
WT
ΔtypA
ΔpstABCS
ΔexbD1
B SynCom
F SynCom
No DNA
ladder
C1 C2 C1 C2 C1 C2 C1 C2 C1 C2 C1 C2 C1 C2
16S rRNA amplification (799F/1192R)
Root179
No Root179
WT
ΔtypA
ΔpstABCS
ΔexbD1
B SynCom
F SynCom
No DNA
ladder
C1 C2 C1 C2 C1 C2 C1 C2 C1 C2 C1 C2 C1 C2
c
Bacteroidetes
Actinobacteria 1
Actinobacteria 2
Bacillales
Alphaproteobacteria
Burkholderiales
Xanthomonadaceae
Acinetobacter
Pseudomonas
typA
exbD1
pstA
pstB
pstC
pstS
Score / max(Score)
1.0
0.8
0.6
0.4
0.2
0.0

Supplementary Fig. 10: Root179 candidate genes involved in root colonization and their conservation profiles across bacteria. (**a**) Root colonization ability of Root179 WT and corresponding knock-out mutants. Colonization is expressed by the number of colony-forming units (CFUs) per milligram of fresh *A. thaliana* roots grown in an agar plate-based system. Each strain was inoculated in 50mL of 1/2MS medium supplemented with MES buffer (final OD600 = 0.0005). 15-20 *A. thaliana* seeds were added to the plates an hour after plate pouring. Plants were harvested at 14dpi. Roots were crushed and dilution series of lyzed roots were plated on 50%TSB square plates for colony counting. *P*-values were obtained from a Dunn's test following a Kruskal-Wallis test. *P*-values between mutants and the WT strain were adjusted with FDR. ns: non-significant, *: $P < 0.05$, ***: $P < 0.001$. (**b**) Validation of a Root179-specific primer pair. Primers were designed in a Root179 gene lacking homologs in other SynCom members using BatchPrimer3 (See methods). DNA deriving from R179 WT or mutant strains (single strains), as well as from the bacterial (B) SynCom (i.e. 83 strains, without Root179) or the fungal (F) SynCom (22 strains) was used together with Root179-specific primers in PCR reactions to validate primer specificity. The primer pair 799F/1192R that targets the bacterial 16S rRNA was used as a control. C1 and C2 indicate two primers concentrations. (**c**) Orthology prediction showing the similarity between Root179 gene families and the most similar gene families in each of the 9 phylogenetic groups from Levy *et al.*, 2018[23]. The similarity score of the best hit in each clade was normalized to the top score obtained for the gene (i.e., most similar gene family). Thus, the lighter the color the more dissimilar the gene families are between Root179 and this phylogenetic group. Source data are provided as a Source Data file.

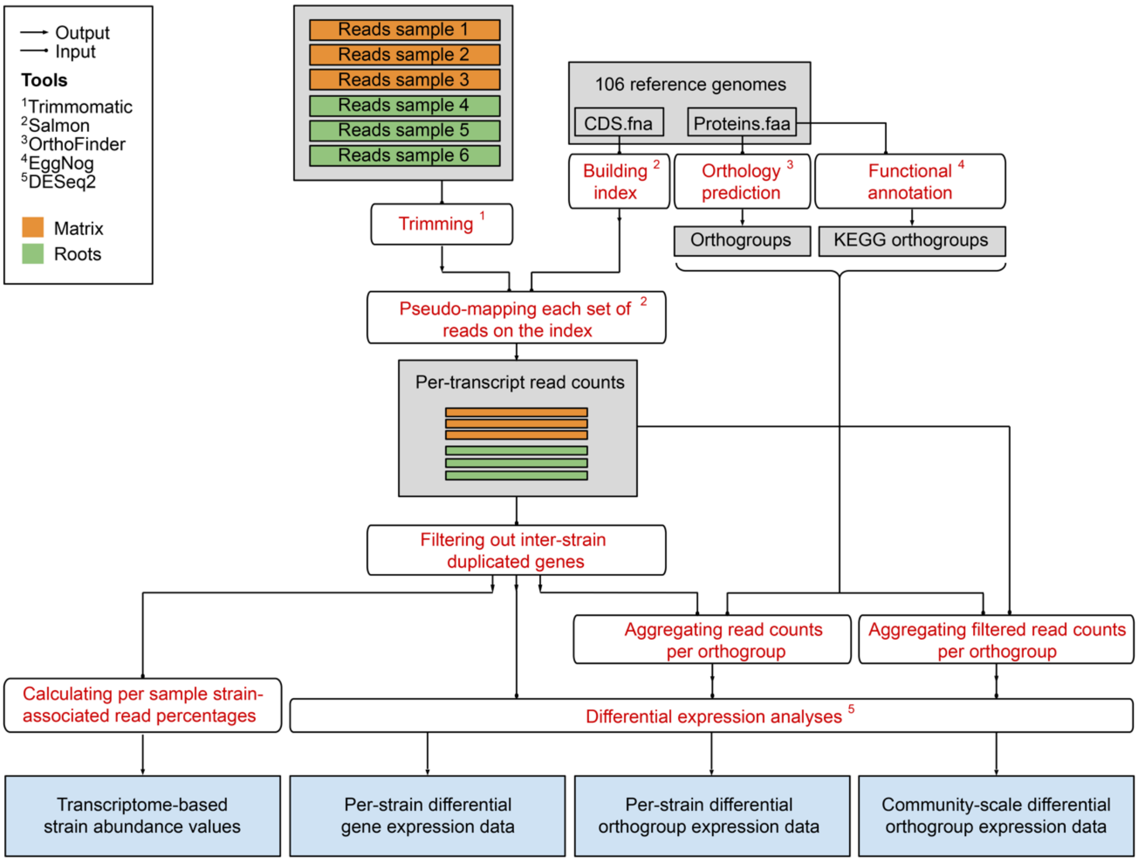

Supplementary Fig. 11: Description of the bioinformatic pipeline used for the analysis of RNA-Seq data. Input and output data are indicated with arrows and tools are indicated with numbers. Matrix and roots samples are indicated in orange and green respectively. Transcripts were trimmed and pseudo-mapped with Salmon against the index comprising the 106 genomes. From this mapping we recovered the non-normalized transcripts quantification (to be able to analyze individual transcriptomes without bias) and filtered-out genes with 100% identity between different bacterial genomes (herein called duplicated genes) to avoid conflicts between strains. These transcript counts were used to calculate strain abundances and per-strain differential expression using DESeq2[2] (individual transcriptomes, gene-level, normalization between samples done in DESeq). Then we aggregated reads per orthogroup for each strain and used orthogroups transcript counts to test for differential expression using DESeq2 (individual transcriptomes, OG-level, normalization between samples done in DESeq2). We then used transcripts without removing 100% identical genes, aggregated the reads per orthogroup for the whole community and tested for differential expression using DESeq2 (community transcriptome, OG-level, normalization between samples done in DESeq2).

Supplementary References

1. Hou, S. et al. A microbiota–root–shoot circuit favours *Arabidopsis* growth over defence under suboptimal light. *Nat. Plants* **7**, 1078–1092 (2021).
2. Love, M.I., Huber, W., Anders, S. Moderated estimation of fold change and dispersion for RNA-seq data with DESeq2. *Genome Biology* **15**, 550 (2014). doi: 10.1186/s13059-014-0550-8.
3. Cantalapiedra, C. P., Hernández-Plaza, A., Letunic, I., Bork, P., & Huerta-Cepas, J. eggNOG-mapper v2: functional annotation, orthology assignments, and domain prediction at the metagenomic scale. *Mol. Biol. Evol.* **38**, 5825-5829 (2021).
4. Rocha, D. J., Santos, C. S., & Pacheco, L. G.. Bacterial reference genes for gene expression studies by RT-qPCR: survey and analysis. *Antonie Van Leeuwenhoek*, ***108***, 685-693 (2015).
5. Alexa, A., Rahnenfuhrer, J.. *topGO: Enrichment Analysis for Gene Ontology*. R package version 2.52.0 (2023).
6. Kanehisa, M. and Goto, S. KEGG: Kyoto Encyclopedia of Genes and Genomes. *Nucleic Acids Res.* **28**, 27-30 (2000).
