# Supplementary material for: Genome-resolved metatranscriptomics reveals conserved root colonization determinants in a synthetic microbiota
Source: Nat Commun. 2023 Dec 13;14:8274. doi: 10.1038/s41467-023-43688-z (PMC10719396; doi:10.1038/s41467-023-43688-z)
Supplement: Supplementary file 3 — Description of Additional Supplementary Files [file 41467_2023_43688_MOESM3_ESM.pdf]

Description of Additional Supplementary Files:

Supplementary Data 1: RNA-based and DNA-based differential microbial enrichment between roots and matrix at class- and genus-level resolution. For both RNA-based and DNA-based microbial abundances, differential abundances between roots and matrix samples were tested using DESeq2 with a two-sided Wald test and a parametric fit. P were adjusted with FDR for each taxonomic level. Abundances were calculated as the sum of all reads belonging to a taxon and these read counts were used for DESeq2 normalization.

Supplementary Data 2: Bacterial genes differentially regulated between roots and matrix. DEGs (roots vs matrix samples) were computed independently for each bacterial strain using DESeq2 with a Wald test. P were corrected with FDR for each bacterial strain separately. Within the table we also provide gene names, the basemean calculated by DESeq2 which represents the corrected average abundance of a transcript, log2FC, predicted gene functions, name and taxonomy of the bacterial strain as well as the orthogroup in which this gene belongs.

Supplementary Data 3: Fungal genes differentially regulated between roots and matrix. DEGs (roots vs matrix samples) were computed independently for each fungal strain using DESeq2 with a Wald test. P were corrected with FDR for each fungal strain separately. Within the table we also provide gene names, the basemean calculated by DESeq2 which represents the corrected average abundance of a transcript, log2FC, predicted gene functions, name and taxonomy of the fungal strain as well as the orthogroup in which this gene belongs.

Supplementary Data 4: PERMANOVA analysis of variance partitioning between transcriptomes. We tested whether the transcriptomes variations between strains could be explained by the compartment and the taxonomy using a multivariate two-sided PERMANOVA based on Bray-Curtis distances calculated from the 20 bacterial strains OGs relative expression.

Supplementary Data 5: GO terms analysis of the top 200 orthologous genes having highest cumulative Log2FC across strains. Go terms analysis testing the enrichment of GO terms top 200 orthologous genes having highest cumulative Log2FC across the 20 bacterial strains. The P were obtained with topGO's Ficher exact test with FDR adjustment.

Supplementary Data 6: List of the microbes used in this study.
