# Supplementary material for: Genome-resolved metatranscriptomics reveals conserved root colonization determinants in a synthetic microbiota
Source: Nat Commun. 2023 Dec 13;14:8274. doi: 10.1038/s41467-023-43688-z (PMC10719396; doi:10.1038/s41467-023-43688-z)
Supplement: Supplementary file 10 — Reporting Summary [file 41467_2023_43688_MOESM10_ESM.pdf]

nature portfolio

Corresponding author(s): Dr. Stéphane Hacquard

Last updated by author(s): Nov 6, 2023

Reporting Summary

Nature Portfolio wishes to improve the reproducibility of the work that we publish. This form provides structure for consistency and transparency in reporting. For further information on Nature Portfolio policies, see our Editorial Policies and the Editorial Policy Checklist.

Statistics

For all statistical analyses, confirm that the following items are present in the figure legend, table legend, main text, or Methods section.

n/a	Confirmed	
☐	☒	The exact sample size (*n*) for each experimental group/condition, given as a discrete number and unit of measurement
☐	☒	A statement on whether measurements were taken from distinct samples or whether the same sample was measured repeatedly
☐	☒	The statistical test(s) used AND whether they are one- or two-sided<br>*Only common tests should be described solely by name; describe more complex techniques in the Methods section.*
☐	☒	A description of all covariates tested
☐	☒	A description of any assumptions or corrections, such as tests of normality and adjustment for multiple comparisons
☐	☒	A full description of the statistical parameters including central tendency (e.g. means) or other basic estimates (e.g. regression coefficient) AND variation (e.g. standard deviation) or associated estimates of uncertainty (e.g. confidence intervals)
☐	☒	For null hypothesis testing, the test statistic (e.g. *F*, *t*, *r*) with confidence intervals, effect sizes, degrees of freedom and *P* value noted<br>*Give P values as exact values whenever suitable.*
☒	☐	For Bayesian analysis, information on the choice of priors and Markov chain Monte Carlo settings
☒	☐	For hierarchical and complex designs, identification of the appropriate level for tests and full reporting of outcomes
☐	☒	Estimates of effect sizes (e.g. Cohen's *d*, Pearson's *r*), indicating how they were calculated

Our web collection on statistics for biologists contains articles on many of the points above.

Software and code

Policy information about availability of computer code

Data collection	No software was used to collect data.
Data analysis	The scripts for the metatranscriptomic analysis pipeline from raw read processing to figure assembly (except differential expression analysis) are available at https://github.com/fantin-mesny/Scripts-from-Vannier-et-al.-2023. The scripts for differential expression analysis and DNA community profiling are available at https://github.com/nathanvannierinrae/Scripts-from-Vannier-et-al.-2023<br>R (version 4.0.3) was used for data transformation, analysis, and visualization. Qiime (v1.9.1, http://qiime.org/) and Usearch (v8.0) were used to process 16S rRNA and ITS read alignment. PyNAST was used to mapped reads against reference genomes.<br>Fungal genomes were assembled with CANU v1.8, and assemblies were polished with Arrow v0.14. Gene prediction was performed with FGENESH v8.0.0. Fungal functional gene annotation was performed using emapper v2 but also different specialized tools: BUSCO2 v5.4.7 (with database ascomycota_odb10) to identify ascomycetal conserved genes likely involved in primary metabolism, SignalP v6.0 to predict genes encoding secreted proteins, EffectorP v3.0 (run in fungal mode on predicted secreted proteins) to identify candidate effector-encoding genes and dbCan2 v4.0.0 to annotate genes encoding carbohydrate active enzymes. Additionally, we used the tool PHIB-Blast (http://phiblast.phi-base.org/) to identify homologs of fungal differentially expressed genes that have been previously characterized and are referenced in database PHIBase v4.15. Functional annotation of the microbial genomes was carried out using the tools emapper v2.1.5 and the EggNog database v5. Orthology predictions were performed with OrthoFinder v2.2.7. Functional annotation of OGs was performed using HMMER v3.1.<br>Quality check and trimming of resulting single-end RNASeq reads was performed using Trimmomatic v0.38, mapping of reads was done with Salmon v0.14.1. Correlation between plant transcriptome was done with Pearson correlation computed using function stats.pearsonr from Python library Scipy.<br>Differential expression of genes, OGs and KEGGs were tested in R 4.0.3 with the package DESeq2 1.30.1 using shrinkage algorithm apeglm v3.14 to correct Log2FC values. Differential relative abundances at the order, class and strain levels were also tested using DESeq2. All volcano

plots were done in R with the package EnhancedVolcano 1.11.3. Pearson correlations between functions' average differential expression and strains abundance in the root compartment were done in R 4.0.3 using the corrplot function from the corrplot package (v0.92). Linear regressions between functions' average differential expression and strains abundance in the root compartment were done in R 4.0.3 using the lm function in the package stats (v4.0.5). Colonization abilities of wildtype, mutant and complemented strains measured by colony counts were tested using a Kruskal-Wallis test followed by a Dunn's test in R, p-values were then adjusted using the p.adjust function in the package stats v3.6.2. To perform Gene Ontology (GO) enrichment analyses was done with the software topGO v2.34.0.

For manuscripts utilizing custom algorithms or software that are central to the research but not yet described in published literature, software must be made available to editors and reviewers. We strongly encourage code deposition in a community repository (e.g. GitHub). See the Nature Portfolio guidelines for submitting code & software for further information.

Data

Policy information about availability of data

All manuscripts must include a data availability statement. This statement should provide the following information, where applicable:

- Accession codes, unique identifiers, or web links for publicly available datasets
- A description of any restrictions on data availability
- For clinical datasets or third party data, please ensure that the statement adheres to our policy

Data availability

The community profiling data generated in this study have been deposited in the ENA database under accession code PRJEB61839 [https://www.ebi.ac.uk/ena/browser/view/PRJEB61839]" (Root samples 16SrRNA: "ERS15411484 [https://www.ebi.ac.uk/ena/browser/view/ERS15411484]", "ERS15411485 [https://www.ebi.ac.uk/ena/browser/view/ERS15411485]", "ERS15411486 [https://www.ebi.ac.uk/ena/browser/view/ERS15411486]"; Soil samples 16SrRNA: "ERS15411487 [https://www.ebi.ac.uk/ena/browser/view/ERS15411487]", "ERS15411488 [https://www.ebi.ac.uk/ena/browser/view/ERS15411488]", "ERS15411489 [https://www.ebi.ac.uk/ena/browser/view/ERS15411489]"; Root samples ITS: "ERS15411490 [https://www.ebi.ac.uk/ena/browser/view/ERS15411490]", "ERS15411491 [https://www.ebi.ac.uk/ena/browser/view/ERS15411491]", "ERS15411492 [https://www.ebi.ac.uk/ena/browser/view/ERS15411492]"; Soil samples ITS: "ERS15411493 [https://www.ebi.ac.uk/ena/browser/view/ERS15411493]", "ERS15411494 [https://www.ebi.ac.uk/ena/browser/view/ERS15411494]", "ERS15411495 [https://www.ebi.ac.uk/ena/browser/view/ERS15411495]"). [add hyperlink for each genome and dataset here and in summary report too]. The genome of Plectospaerella_cucumerina_MPI-CAGE-AT-0143 generated in this study have been deposited in the ENA database under project ID PRJEB61839 [https://www.ebi.ac.uk/ena/browser/view/PRJEB61839] under sample ID ERS15396667 [https://www.ebi.ac.uk/ena/browser/view/ERS15396667]. The genomes of Fusarium_oxysporum_MPI-CAGE-AT-0013 (Fusarium13), Fusarium_redolens_MPI-CAGE-CH-0216 (Fusarium216), Fusarium_oxysporum_MPI-CAGE-CH-0226 (Fusarium226), Fusarium_equiseti_MPI-CAGE-CH-0233 (Fusarium233) have been deposited in the ENA database under accession code PRJEB50298 [https://www.ebi.ac.uk/ena/browser/view/PRJEB50298]. The other genomes have been previously published and were obtained from "TAIR [http://arabidopsis.org]" (A. thaliana), "At-Sphere [http://at-sphere.com]" (82 bacteria), "Mycocosm [https://mycocosm.jgi.doe.gov]" (17/22 fungi). The raw RNAseq reads, as well as the reference CDS used for mapping are available at GEO, under accession "GSE231841 [https://www.ncbi.nlm.nih.gov/geo/query/acc.cgi?acc=GSE231841]". Source data are provided with this paper. All the data generated in this study are provided in the Supplementary Information/Source Data file.

Research involving human participants, their data, or biological material

Policy information about studies with human participants or human data. See also policy information about sex, gender (identity/presentation), and sexual orientation and race, ethnicity and racism.

Reporting on sex and gender	Not applicable
Reporting on race, ethnicity, or other socially relevant groupings	Not applicable
Population characteristics	Not applicable
Recruitment	Not applicable
Ethics oversight	Not applicable

Note that full information on the approval of the study protocol must also be provided in the manuscript.

Field-specific reporting

Please select the one below that is the best fit for your research. If you are not sure, read the appropriate sections before making your selection.

☒ Life sciences ☐ Behavioural & social sciences ☐ Ecological, evolutionary & environmental sciences

For a reference copy of the document with all sections, see nature.com/documents/nr-reporting-summary-flat.pdf

Life sciences study design

All studies must disclose on these points even when the disclosure is negative.

Sample size	No sample-size calculation was performed to predetermine the number of samples. Rather, efforts were made to ensure large replicate numbers per experiment. Each experiment except RNA-seq was designed to contain at least 10 technical replicates per treatment for statistics, and the experiments were replicated to ensure reproducibility.

	For RNASeq experiments, the roots of all plants from three FlowPots were combined for a total of 9 plants per sample and 6 total samples, 3 from matrix and 3 from root. For root colonization assays in agar plates, 3 plates with 10 plants each were used per condition. For liquid growth of mutant strains 5 replicates growth curves per condition. For root colonization assay with SynComs in flowpots 12 replicates were done per condition, consisting of 2 independent biological batches of 6 technical replicates.
Data exclusions	No sample for 16S rRNA or ITS sequences and RNAseq were excluded. Few samples where excluded in subsequent mutant experiments for plants that did not germinate . For qPCR analyses few samples were excluded for which no Ct values were obtained.
Replication	Initial RNAseq experiment was performed only once because of experiment financial cost but subsequent experiments with mutants contained independent replicates to confirm reproducibility. All findings from replicate experiments were consistent and confirmed. The mutants root colonization experiment was performed in two independent experiments. The complementation mutants root colonization experiment was performed in three independent experiments. The mutant flowpot reconolonization experiment was performed over three independent experiments. The second RNA-seq experiment comparing bacterial transcriptomes in soil with and without fungi was performed once, also because of financial cost.
Randomization	All agar plates and box for flowpots experiment was distributed at random into the growth chambers, and randomly shuffled every two to three days to minimize location-based effects. Samples were collected randomly for each condition in all experiments.
Blinding	All samples were not annotated with condition names but with a numbering, therefore avoiding condition identification during sampling and all subsequent molecular biology processing.

Reporting for specific materials, systems and methods

We require information from authors about some types of materials, experimental systems and methods used in many studies. Here, indicate whether each material, system or method listed is relevant to your study. If you are not sure if a list item applies to your research, read the appropriate section before selecting a response.

Materials & experimental systems

n/a	Involved in the study
☒	☐ Antibodies
☒	☐ Eukaryotic cell lines
☒	☐ Palaeontology and archaeology
☒	☐ Animals and other organisms
☒	☐ Clinical data
☒	☐ Dual use research of concern
☐	☒ Plants

Methods

n/a	Involved in the study
☒	☐ ChIP-seq
☒	☐ Flow cytometry
☒	☐ MRI-based neuroimaging
